# Transverse dielectrophoretic-based DNA nanoscale confinement

**DOI:** 10.1038/s41598-018-24132-5

**Published:** 2018-04-13

**Authors:** Sara Mahshid, Jia Lu, Abrar A. Abidi, Robert Sladek, Walter W. Reisner, Mohammed Jalal Ahamed

**Affiliations:** 1 McGill University, Department of Bioengineering, Montreal, H3A 0E9 Canada; 2 McGill University, Department of Physics, Montreal, H3A 2T8 Canada; 3 McGill University, Department of Human Genetics, Montreal, H3A 0C7 Canada; 4 University of Windsor, Department of Mechanical, Automotive and Materials Engineering, Windsor, N9B 3P4 Canada

## Abstract

Confinement of single molecules within nanoscale environments is crucial in a range of fields, including biomedicine, genomics, and biophysics. Here, we present a method that can concentrate, confine, and linearly stretch DNA molecules within a single optical field of view using dielectrophoretic (DEP) force. The method can convert an open surface into one confining DNA molecules without a requirement for bonding, hydrodynamic or mechanical components. We use a transverse DEP field between a top coverslip and a bottom substrate, both of which are coated with a transparent conductive material. Both layers are attached using double-sided tape, defining the chamber. The nanofeatures lie at the “floor” and do not require any bonding. With the application of an alternating (AC) electric field (2 V_p-p_) between the top and bottom electrodes, a DEP field gradient is established and used to concentrate, confine and linearly extend DNA in nanogrooves as small as 100-nm in width. We also demonstrate reversible loading/unloading of DNA molecules into nanogrooves and nanopits by switching frequency (between 10 kHz to 100 kHz). The technology presented in this paper provides a new method for single-molecule trapping and analysis.

## Introduction

DNA linearization plays an important role in achieving scientific insights into polymer physics and shows great potential in DNA sequencing and mapping^1^. Stretching DNA on an optically accessible surface allows direct visualization and acquisition of contextual information for single extended DNA molecule^2–4^. As a result of the rapid development of semiconductor-based micro/nanofabrication technology, fabrication of nanofeatures as small as tens of nanometers wide is feasible, allowing researchers to trap, probe, linearize, and visualize single DNA molecules in a single field of view^4^. Recently, on-chip analysis of long (gigabase-scale) genomes extracted from a single-cell was demonstrated^5–10^. Despite these demonstrations, nanofluidic-based technologies still face challenges in loading DNA into nanofeatures, mainly due to the complicated fluidic network that is required to interface between micro- and nano-scale fluidic features. There is a high demand for simple, reliable and cost-effective means of on-chip loading and linearizing DNA that avoids fragmentation of long molecules.

Technologies for on-chip DNA linearization can be grouped into three categories: (1) fixed confinement, (2) tuneable confinement and (3) free surface stretching. In the first method, which is widely used in nanofluidic devices, DNA is hydrodynamically loaded into a fixed nanoconfined space. When a DNA molecule is confined in a space with a dimension below its free solution gyration radius, it will stretch linearly^4,11–13^. In such approaches, DNA is initially pipetted into the microfluidic side of the chip and then threaded into the nanofluidic regions by applying either a mechanical force (pneumatic or hydrostatic) or an electrokinetic force^2,9,14–18^. In the micro-nano fluidic approach, the abrupt increase in dimension from the macro- to the nano-scale is associated with sharp increase in free energy and a large free energy barrier, preventing molecules from entering the nanoconfined region. Overcoming this barrier requires high hydrodynamic pressure at the micro-nano interface, which can potentially lead to DNA fragmentation. To address this loading issue, various interface improvements have been developed. One method involves using varying and gradient geometries (such as a funnel) to create a longer entrance length at the micro/nano inlet^19–23^. Alternatively, DNA can be deposited and stretched on a modified device surface^24^.

In the second approach, DNA is loaded and stretched inside a space with tuneable varying nanoconfinement. This tuneable confinement transforms a micro-scale chamber into a nanoscale chamber, which can be achieved by either deforming a thin membrane from the top or deforming a channel wall from the side^25–29^. Combined with nanofabrication technologies, it can be used to create devices that can perform high-throughput capture of DNA and other macromolecules^26^. Such methods require specialized equipment and a mechanical force to provide the deformation mechanism. Moreover, due to the radius of curvature of the curved surface, the confinement varies from the convex surface to the centre of the confinement area, creating only a localized degree of confinement. Another approach, mechanical collapse of triangular elastomeric nanochannels for stretching DNA offers a robust method for confinement that avoids creating a large confinement gradient^30^.

In the third strategy, DNA is stretched without applying any physical confinement, on a free surface or in a free solution with an optical or magnetic trap. In this method, no physical confinement is required. Stretching without confinement is achieved by applying either an electric field (electro stretching)^31,32^ or a hydrodynamic force (flow stretching)^33,34^ or by using an optical trap^35^. Stretching DNA on the free surface using molecular combing has been pursued extensively and has led to many different methods of DNA combing and stretching^24,36–39^. This process primarily uses forces exerted by a receding capillary meniscus. The advantage of this approach is that it does not require nanofabrication. Several applications have been developed using this method, such as the extension of long genomic DNA (gigabase-scale). In most free surface based methods, it is challenging to create a reproducible molecular extension, as the surface bound molecule can be overstretched and is free to deviate from a completely straight trajectory. In contrast, in a nanochannel approach, the molecule’s equilibrium conformation is altered due to self-avoidance interactions, leading to a linear un-scrolling of the genome along the nanochannel while the molecule remains free (e.g. not immobilized to the channel surface). The nanochannel approach allows for continuous measurement while keeping the DNA constantly in the field of view for extended periods.

**Figure 1:**
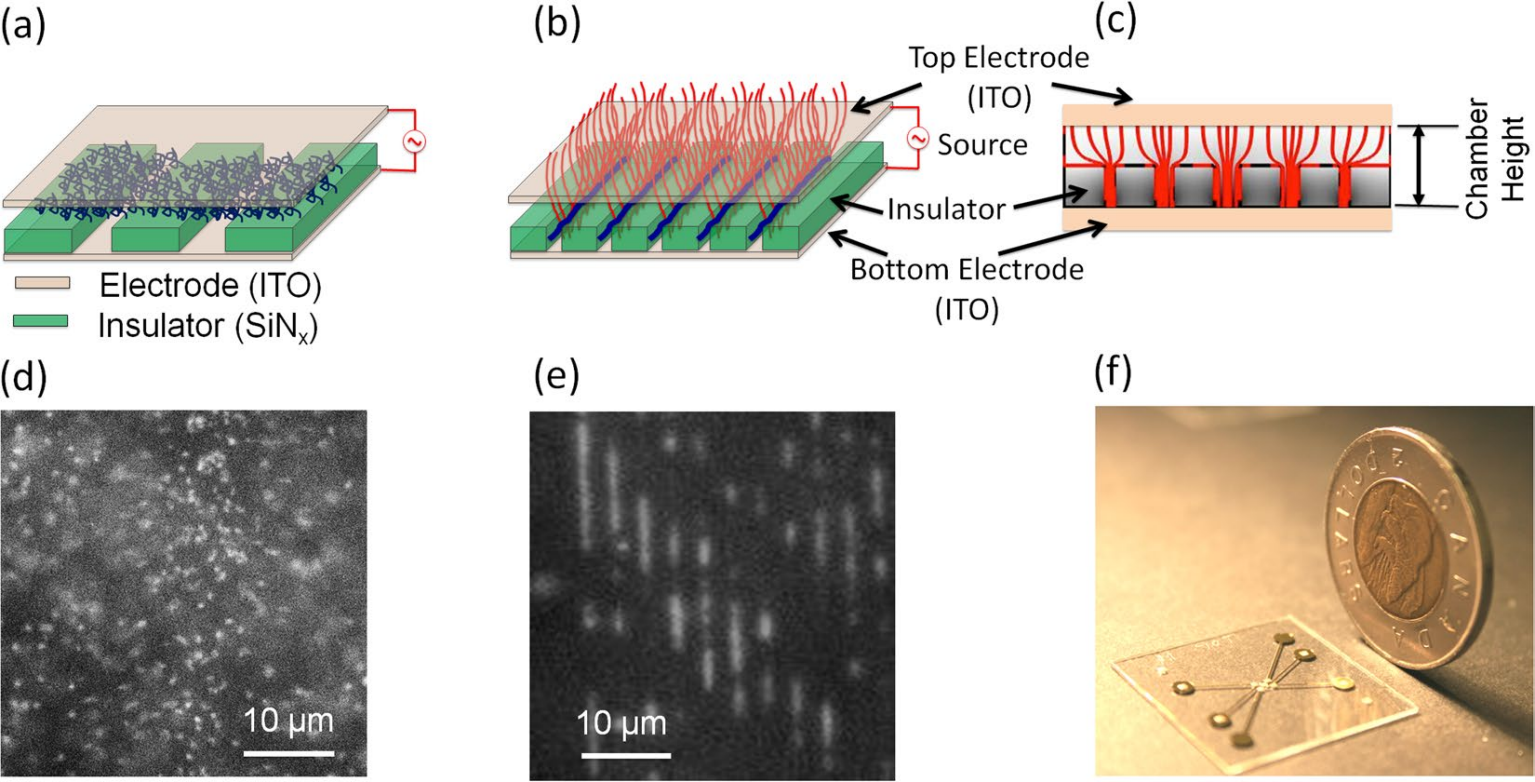
Schematic of the device shows (**a**) two parallel plate electrodes separated by a spacer defining the chamber. DNA (blue) is located inside the chamber in a free solution before applying electric field; (**b**) nanoscale confinement of DNA molecules with an applied AC field creating a transverse dielectrophoretic force; (**c**) numerical model of the chamber (with vertical chamber spacer height of 30 μm) showing the transverse electric field lines (red) from the top to bottom electrode; (**d**,**e**) fluorescence image showing DNA molecules inside the chamber before and after applying the electric field. (**e**) With a transverse DEP force, DNA is confined and linearly stretched inside 300-nm channels. (**f**) Image of a fused silica prototype.

An ideal DNA stretching technology would overcome the limitations of classical nanofluidics-based technologies but be as easy to implement as surface stretching. Accordingly, it would be beneficial to provide a new technological option that leverages the benefits of nanoscale confinement to provide a platform that can confine a large number of molecules within a single field of view, with simple fluidics integration and operation. At the same time, the technology would allow optical observation of single molecules within an open and uniform environment without requiring hydrodynamic forces, mechanical components, or the need for a very thin (nanoscale) vertically bonded device. In this paper, we present such a method. Our method utilizes a combination of electric force and nanogrooves to linearize single DNA molecules. The method can transform one simple glass slide into a micro-scale flow cell (through double-sided tape), and no hydrodynamic or mechanical forces are required to isolate and extend DNA molecules (Figure 1a). At the beginning of device operation, DNA molecules are in free solution (Figure 1a). With the application of a dielectrophoretic (DEP) field, our method traps, confines, and linearizes DNA (Figure 1b). Our method is based on implementation of a dielectrophoretic force to load DNA into a nanoconfinement from the top. Figure 1c shows the direction of the field lines between the top and bottom electrodes. Before applying an electric field, DNA is in free solution (Figure 1d, fluorescence image of DNA in free solution). With the application of a DEP field, DNA is linearized inside the nanogrooves that lie on the bottom plane (Figure 1e). In our method, we overcome the limitation of surface-based electro-stretching by maintaining DNA inside a region of fixed confinement. We overcome the limitations of classical nanofluidic-based stretching by obviating the need for any pressure-driven flow, external mechanical force or bonding. We load DNA into the confinement from the top, without the need of any mechanical force, overcoming complex mechanical loading. While DEP-based micro/nanofluidic molecule manipulation^40–43^ and DNA electro-stretching is a well-established field that has been explored by many researchers for decades^44–49^, it has never been implemented to confine DNA in a nanoscale environment. Here, for the first time, we apply a transverse DEP on a transparent surface with embedded nanotopographies for DNA trapping and linearization. Our method not only linearizes DNA but also concentrates DNA molecules.

In classical micro/nanofluidic methods, loading a biomolecule solution into the device and moving it to the centre of the device is challenging and time-consuming. By contrast, in our approach, the top and bottom layers of the device are joined by a tape spacer, opening the chamber to fluid flow (Figure 1a). The advantage of our open-surface design is flexibility in DNA loading, with adjustable loading of DNA into nanopatterned features like nanogrooves and nanopits made possible by introducing a transverse DEP force into our system. We create the transverse DEP force by applying an alternating electric (AC) field between parallel electrodes on the top and bottom device surfaces. The DEP force can be controlled by varying the frequency of the applied electric field. In the next section, we demonstrate DNA molecule loading and linearization using this approach.

## Results and Discussion

Dielectrophoresis (DEP) is a phenomenon that occurs with a non-uniform electric field, in which an electrically neutral particle feels a force due to the interaction between the electric field and the particle’s induced dipole moment. The direction of the DEP-induced particle motion depends on the particle’s polarizability relative to the suspending medium, which in turn depends on the frequency of the applied electric field. The particles may move towards the higher electric field (called a positive DEP effect) or away from the field (negative DEP effect). Switching between positive and negative DEP can be achieved by adjusting the frequency of the electric field. In our design, an array of electrodes is located at the bottom surface (Figure 1a), where DNA is trapped using a DEP force. To predict the device performance and optimize the device design and the actuation signal, we developed a numerical simulation. To predict the device performance and optimize the device design, we utilized finite element simulation to model the positive and negative DEP fields (COMSOL Multiphysics 5.2a).

**Figure 2:**
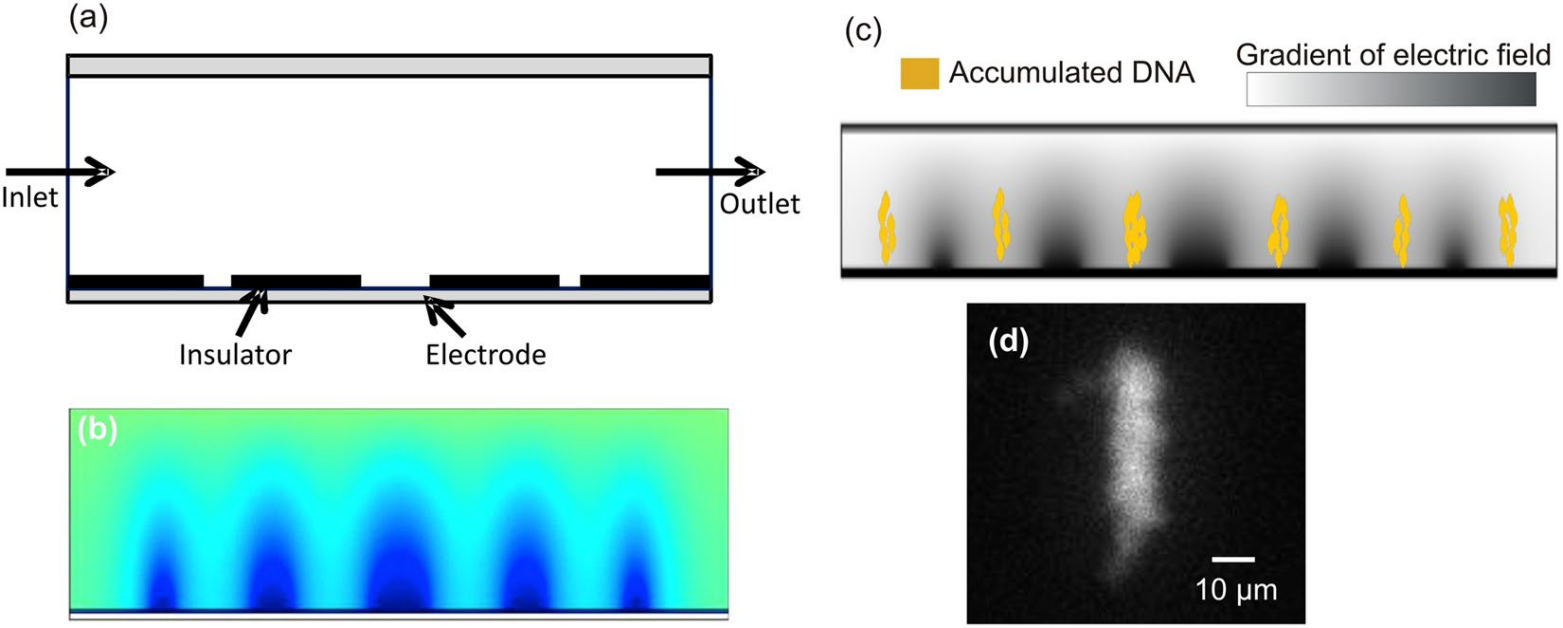
Schematic diagram showing the model geometry with electrodes, inlet/outlet, insulator and channel wall; (**b**) surface map showing the normalized electrical field strength for the applied voltage of 2 V; (**c**) simulation showing the accumulation of molecules due to the negative DEP field. The surface map of electric field gradient (grey) is superimposed with particle (yellow) accumulation and (**d**) fluorescent image showing DNA concentration of λ-Phage DNA with application of DEP force.

A simplified 2D model was developed, representing the cross-section of the device (Figure 2a). In our simulation, we considered DNA molecules as spherical particles. In experiments, we observed that in free solution (with 30 µm chamber vertical height), DNA coagulated at a size of approximately 4–5 µm. In our numerical simulation, for simplicity, we modelled ***λ***-Phage DNA molecules as spherical particles with a diameter of 4 µm. In free solution and in the native state, ***λ***-Phage DNA forms a coil that is approximately 2–4 µm in diameter^50^. In our device, the gap between the top and bottom coverslips is 30 µm; the gap is larger than the DNA coil size, consequently, DNA is not stretched in the free solution. Therefore, the spherical assumption is a reasonable approximation and has been used in similar modelling^51^. The simulation objective is to identify possible locations of particle concentration in free solution in our design during DEP application. This particle tracking simulation solves for three physical quantities: electric current, creeping flow velocity and particle trajectory. The conductivity of 1× TBE is assumed to be 859 μS/cm. The dielectrophoretic force F_DEP_ on a spherical particle is given by^52^

1 $$F=2\pi {r}_{p}^{3}k\nabla {|{E}_{rms}|}^{2}$$

where

2 $$k={{\varepsilon }}_{0}Re\{{{\varepsilon }}_{f}\}Re\{\frac{{{\varepsilon }}_{p}-{{\varepsilon }}_{f}}{{{\varepsilon }}_{p}+2{{\varepsilon }}_{f}}\}$$

where *ɛ*_*p*_ and *ɛ*_*f*_ represent the permittivity of the particle and the fluid, respectively. The radius of the particle is *r*_*p*_, and *E*_*rms*_ is the root-mean-square amplitude of the applied alternating electric field. Here, *ɛ* is the complex permittivity given by

3 $${\varepsilon }={{\varepsilon }}_{real}-j\frac{{\sigma }}{2{\pi }{\nu }}$$

where *ε*_*real*_ is the real component of the permittivity, *σ* is the electric conductivity and *ν* is the frequency of the alternating field.

In the time domain equation for the DEP force (equation 1), the difference in permittivity between the fluid and particle determines the sign of the equation, and thus the direction of the DEP force. When the sign of this difference changes from positive to negative, the direction of the force also changes. The difference and sum of permittivity between the particle and medium can be described by the Clausius-Mossotti function^52^. Figure 2b shows a surface map of the electric field distributed across the chamber, with darker regions representing higher field strength. DNA molecules can be either captured by the electrode (positive DEP) or repelled from the high electric field and then accumulate on the insulator. Larger electrodes have less concentrated electric fields. The trajectories of the DNA particles were calculated by solving the equation of motion for each particle using Newton’s second law as:

4 $$\frac{d}{dt}({m}_{p}V)=F$$

where *m*_*p*_ and *V* are the particle mass and velocity, respectively, and F is the total force experienced by the particle. There are three forces that act on the particle when it is suspended in liquid: the DEP force, fluid viscous force, and Brownian motion. The effects of the gravitational force and buoyant force are negligible and combining the three net forces gives the equation below^53^:

5 $$F=\frac{1}{{{\tau }}_{p}}{m}_{p}(U-V)+{\varsigma }\sqrt{\frac{12\pi {k}_{B}\mu T{r}_{p}}{\triangle t}}+2\pi {r}_{p}^{3}k\nabla {|{E}_{rms}|}^{2}$$

where *k*_*B*_ is the Boltzmann constant, *T* is temperature, *µ* is fluid viscosity, Δ*t* is the time step, *U* is fluid velocity, *r*_*p*_ particle radius and *τ*_*p*_ is related to the response time. The first term in the equation for net force is the drag force, the second term is Brownian force, and the third term is the DEP force. Our model describes the particle movement due to the DEP-induced force. The flow is assumed to be a low Reynolds number Stokes flow. The flow of particles is due to the DEP-induced force, which is an electrokinetic flow in the horizontal direction. In the transverse DEP, there is a flow in the vertical direction.

This transverse flow effect was reported by Pethig *et al*.^54^, who showed that at low frequencies (<100 kHz), particles collect on the upper surfaces of electrode structures. In contrast, the molecules close to the bottom electrode experience a regular DEP effect. Based on the applied frequency, they either experience a positive DEP (towards the increasing electric field) or a negative DEP (towards decreasing electric field). We observed similar phenomena in our simulation. At low frequencies, molecules collected at the upper electrode surface, which is not desirable, as the nanogrooves are located at the bottom surface. However, at frequencies above 100 kHz, we observed that DNA collects near the bottom surface. Figure 2c shows the simulation results for the accumulation of DNA molecules on the bottom electrodes of different dimensions. In addition to the effect of varying frequency, our simulation shows that the size of the electrodes plays a crucial role in determining the location and degree of particle concentration. In particular, decreasing the electrode width leads to more localized accumulation around the electrode with a greater molecule concentration at the electrode surface. In Figure 2d, particles are accumulated between the bundled DEP field lines.

**Figure 3:**
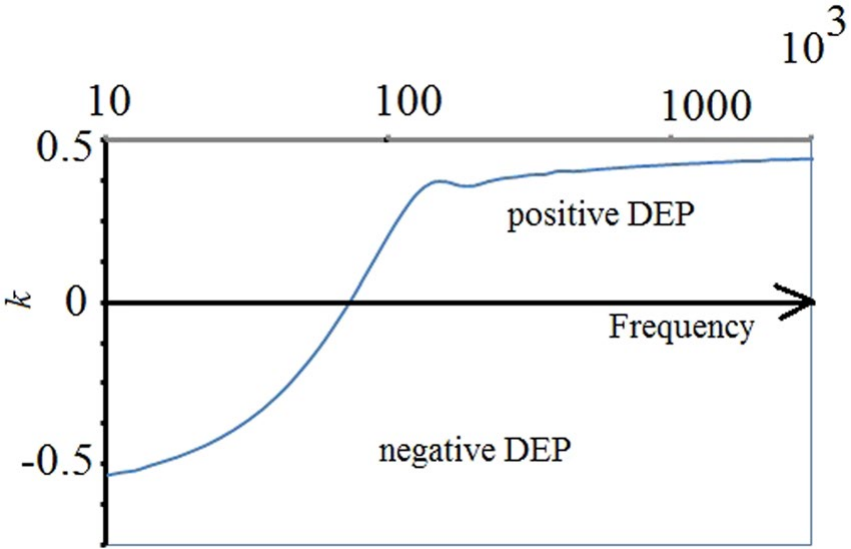
Clausius-Mossotti function *k* (equation 2) plot showing cross-over between positive to negative DEP and the crossover frequency.

The plot of the Clausius-Mossotti function (Figure 3) can be used to predict the effect of frequency on the DEP force, showing cross-over between positive to negative DEP. Note that for our initial geometry of 30 μm, the crossover frequency from positive to negative DEP was estimated to be slightly less than 100 kHz.

The simulation results provided estimates for key design and operating parameters, such as the operating frequency and voltage, for a given electrode gap and solution medium. To this end, we performed experiments to verify the concept and show applications of the method for trapping DNA in the nanogrooves and nanopits.

### DEP-assisted accumulation and concentration of DNA molecules

Using nano-groove electrode structures, the DEP effect can be used to perform single molecule manipulation, such as DNA extension, as well as DNA concentration. In order to build up high concentrations of molecules near the nanogrooves, to ensure high-throughput molecule linearization, we first utilize the negative DEP effect to pre-concentrate DNA near the nanogrooves. When a DNA sample at low concentration is loaded into the device, the molecules must be transported and concentrated close to the nanogrooves. DNA molecules experience DEP-induced-force when exposed to an AC electric field. The strength of the field is relevant to the surface area of the electrodes and the applied frequency^49^.

**Figure 4:**
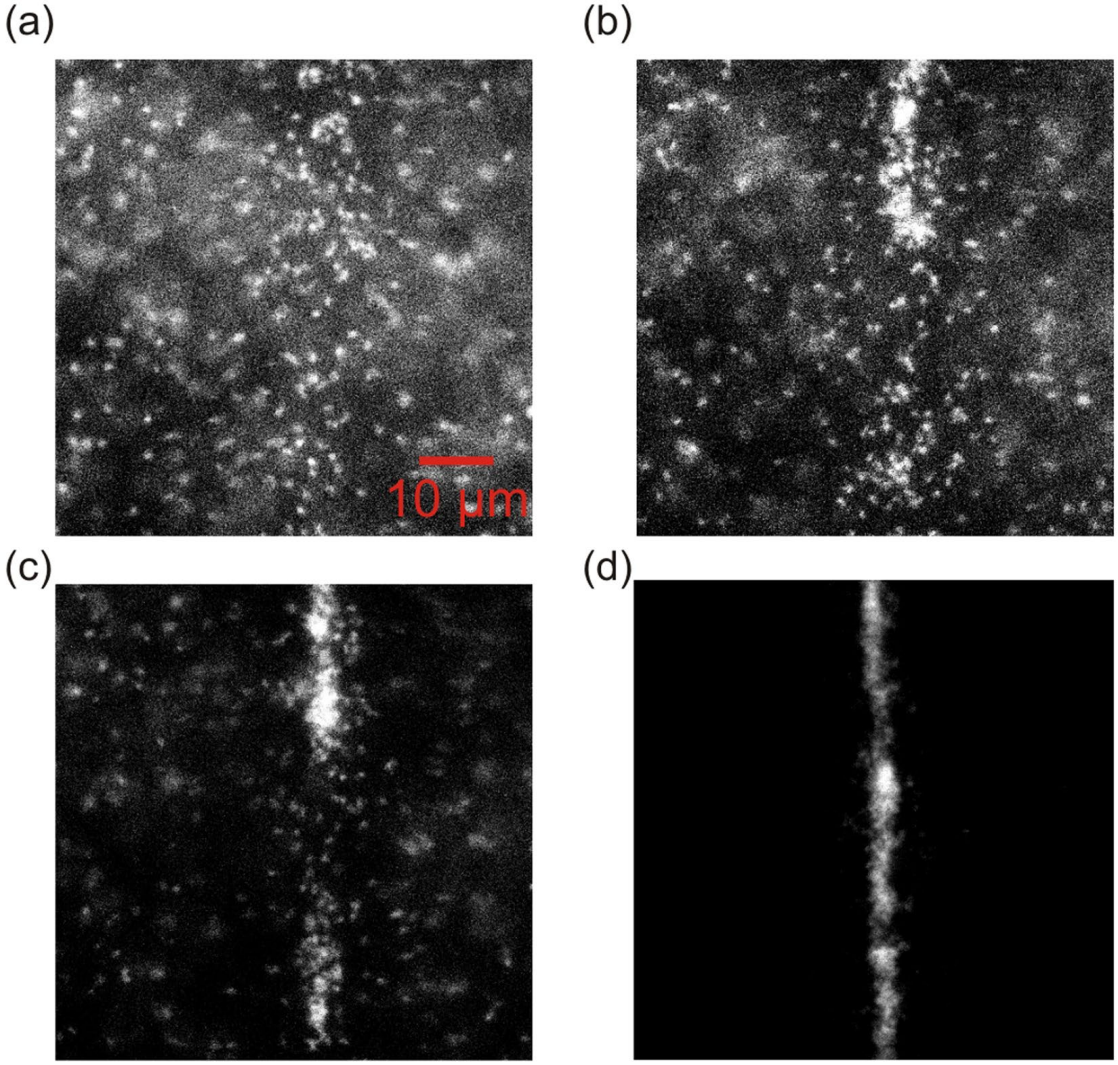
(**a**–**d**) DNA build-up along an electrode upon ramping frequency from 10–200 kHz (2 Vpp). (**a**) DNA in free solution starts to move; (**b**) upon application of DEP, molecules start to move towards the electrode (in image center); (**c**,**d**) molecules eventually become highly concentrated along the electrode.

To pre-concentrate molecules, we implemented DEP-assisted concentration of DNA molecules using microscale-patterned electrodes. The arrays of transparent ITO (Indium Tin Oxide) electrodes are divided by silicon nitride insulators. The DNA molecules are either captured by the ITO electrode (positive DEP) or repelled from the high electric field and accumulated on the insulator. For example, at 2 V_P-P_ , at approximately 10 kHz, we observed the onset of a negative DEP, while at 2 V_P-P_ , at approximately 100 kHz, we observed a switch to a positive DEP, as shown in Figure 4. Figure 4(a–d) shows the time sequence of the DNA concentration using the frequency. When an electric field is applied with frequency between 20–30 kHz, the DNA starts to move towards the electrode as shown in Figure 4a,b; and when the frequency reaches approximately 100 kHz, the DNA accumulates along the rectangular electrode (Figure 4d).

**Figure 5:**
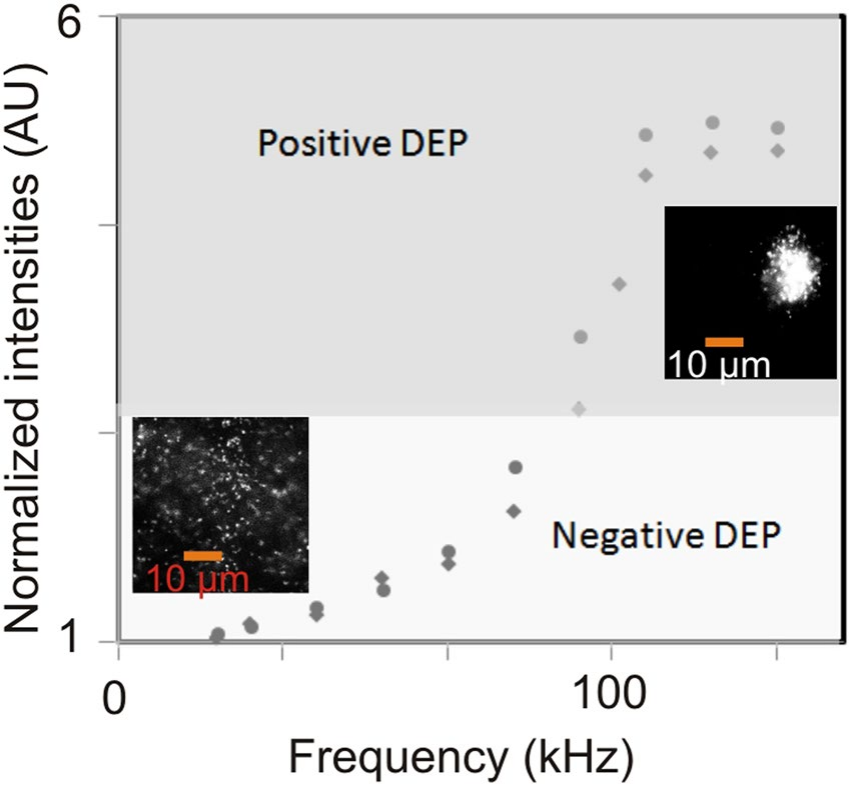
Normalized electrode intensity versus frequency showing molecule focusing along the electrode.

Figure 5 shows concentration of DNA molecules using DEP with increasing frequency. In order to quantify the effect of varying DEP frequency on DNA concentration in the electrode vicinity, we first integrate the DNA intensity over a rectangular region aligned and superimposed on the electrode. We then compute the ratio of this integrated intensity to the integrated intensity prior to the application of DEP. We find that this measure of DNA concentration, which we call the Normalized Electrode Intensity, has a sharp transition at around 100 kHz associated with the onset of positive DEP. At frequencies below the cross-over frequency of 100 kHz, we observed negative DEP effects, where DNA moved away from electrodes (Figure 5); at a higher frequency, the positive DEP effect concentrates DNA along higher electric fields. DNA accumulation saturates when the frequency exceeds 500 kHz.

### DNA nanochannel loading and linearization

**Figure 6:**
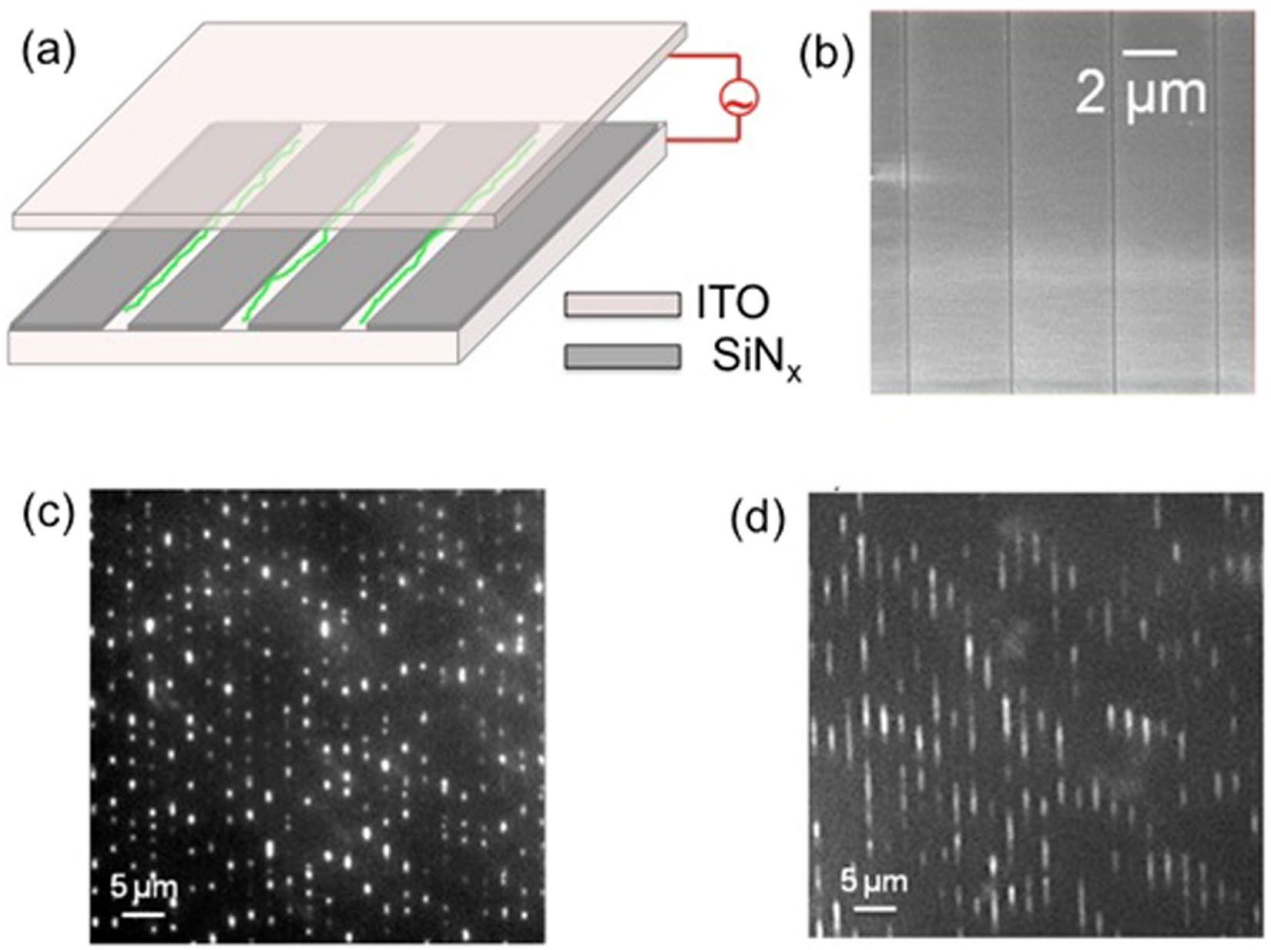
(**a**) Schematic of DEP-assisted DNA (green) confinement and linearization on nanoscale grooves; (**b**) SEM image of nanogroove electrodes. Fluorescent image of λ-phage DNA on (**c**) 1-μm and (**d**) 300-nm nanopatterned electrodes showing parallel confinement and extension.

We demonstrate DEP-based DNA loading in an open-plane nanochannel (nanogroove) by loading λ-phage DNA into nanogroove structures. DNA molecules transform from a coiled to an extended conformation when loaded into nanogrooves. Figure 6 shows the concept (Figure 6a,b) and experimental images (Figure 6c,d) for DNA loaded into 300-nm and 1-μm grooves. The method is reversible, which means that it is possible to simultaneously load and unload DNAs from nanofeatures by switching between positive and negative DEP effect (video SV1 showing loading in 300 nm channel). Loading/unloading can be achieved in a fraction of a second, allowing high-speed DNA loading at the nanoscale. Figure 6c,d shows images of DNA loaded into 1-μm and 300-nm grooves, respectively. We achieved reversible loading in 300-nm nanochannels, down to as low as 100-nm nanochannels.

At high frequencies (>100 kHz), DNA molecules experience a positive DEP force, which drives them towards the field lines and initiates loading. Above 100 kHz, DNA molecules are trapped in regions with strong electric fields and are loaded inside the nanogrooves. Likewise, DNA molecules are repelled from the field lines by negative DEP forces at low frequencies (<10 kHz). With a 30 µm spacing between the electrodes, we found that reversible loading of DNA into nanogrooves can be achieved by tuning the frequency between 10 kHz to 100 kHz.

**Figure 7:**
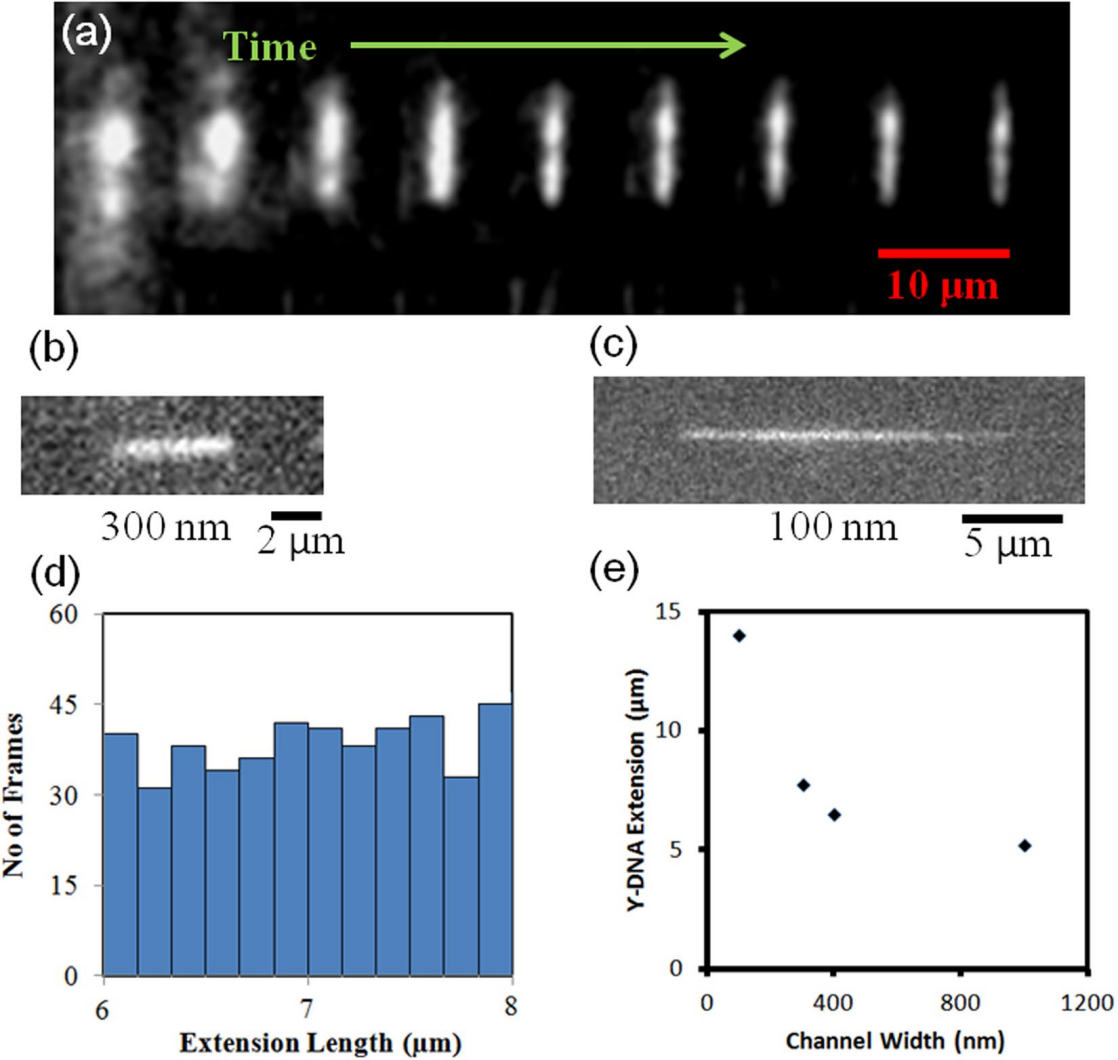
(**a**) Time series images of λ-phage DNA extension in nanoscale confinement with DEP force. (**b**) and (**c**) λ-phage DNA confinement and extension on (**b**) 300-nm and (**c**) 100-nm electrodes. (**d**) Probability density of λ-phage DNA length at several time frames, and (**e**) λ-phage DNA extension in different electrode dimensions.

Figure 7a shows a time sequence of DNA loading in the nanochannel. In these images, the objective is focused on the surface containing the nanogrooves. As the frequency increases to 10 kHz, the molecules are brought closer to the nanogrooves until they are immobilized inside the nanogrooves and extended (Supporting Video SV1). We attribute this phenomenon to a positive DEP response, which should accumulate DNA in the grooves.

**Figure 8:**
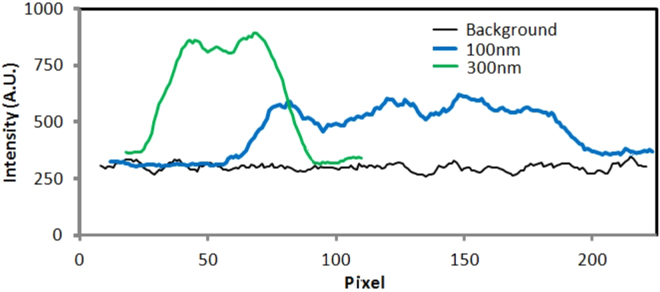
Intensity plot across linearized DNA for 300-nm and 100-nm channel and background signal intensity.

**Figure 9:**
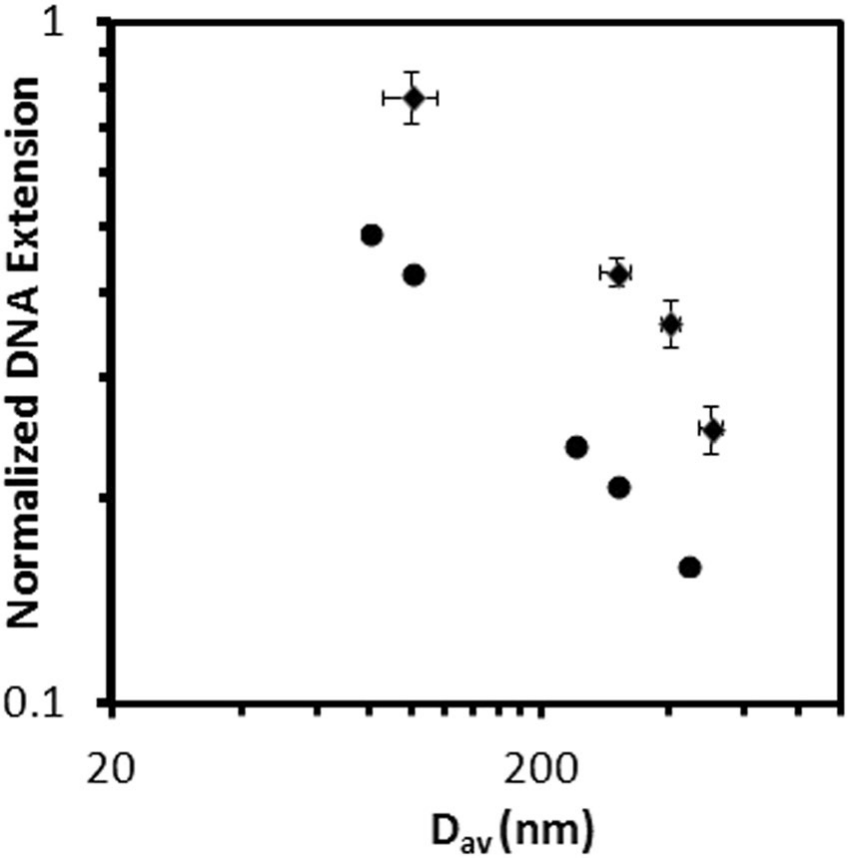
Comparison of the DNA extension length with the geometric average dimensions plotted in log-log scale. Circles are data of conventional confinement extension from the literature^55,56^, and diamonds are DEP-based extension.

We hypothesize that the stretching effect is a combination of the DEP force (which immobilizes DNA in the nanogrooves) and confinement in an effective 1D structure formed from the DEP potential and channel sidewalls. Figure 7(b,c) show DNA linearized in 100-nm and 300-nm nanochannels, respectively. The histogram plot (Figure 7d) shows consistency in the extended length with time. The extension is governed by confinement scaling laws and decreases with increasing nanogroove size (Figure 7e). The raw intensity signal plot across the extended DNA for the 300-nm and 100-nm channels is shown in Figure 8, along with the background signal. Figure 9 compares DNA extension obtained using our system to values in the literature with similar nanochannel geometry and fused-silica substrate working with λ-DNA reported in^55,56^ DNA extension was normalized using a total contour length of 18.63 μm. As seen in the figure, the extension in purely confinement based systems with similar dimensions has lower extension than in our system. The difference in extension supports our hypothesis that DEP helps stretch the DNA.

### DNA loading in nanopits

**Figure 10:**
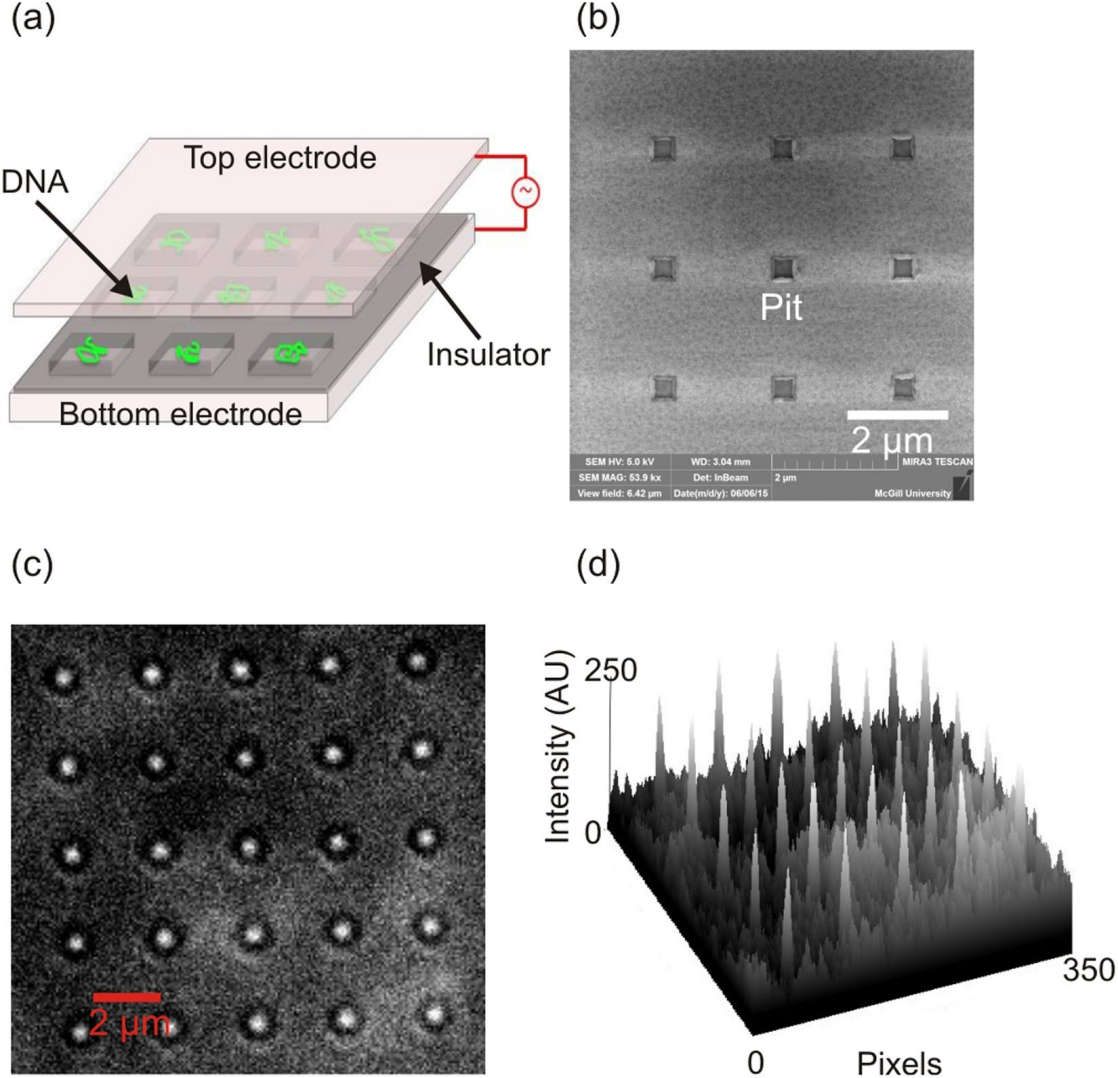
(**a**) Schematic diagram of DEP-assisted DNA confinement on nanoscale pits, (**b**) SEM image of the device with nanopatterned pits. (**c**) Fluorescence image showing DEP-assisted λ-phage DNA confined in nanopits (bright spots). (**d**) The intensity profile shows sharp peaks at confined DNA molecules inside nanopits.

DNA loading and trapping in nanocavities is important for various applications including commercial sequencing applications^57^. Loading and trapping of DNA in nanopits is important not only for polymer physics studies but also for DNA sequencing^3,15,58,59^. Some sequencers are based on conventional nanofluidic flow-based DNA loading methods. A combination of nano-pores and nanowells (zero mode waveguide) has been used to demonstrate efficient length-independent DNA loading^60,61^. Our method can also be applied to rapidly and reversibly load DNA into nanopits; however, in contrast our method uses an open surface and dielectrophoretic force to load DNA in pits (Figure 10). We use single step e-beam lithography to create nanopatterned pits by etching silicon nitride and exposing the ITO electrode to create square arrays of nanopits (SEM image in Figure 10b). Figure 10c shows λ-phage DNA being loaded onto 300 nm pits with uniform optical signals being detected from the pit wells (Figure 10d).

## Conclusion

In this work, we present a new technology that addresses the challenges of conventional micro/nanofluidic based DNA linearization by introducing a dielctrophoretic force for reversible DNA loading/unloading and linearization inside nanofeatures. We demonstrate the application of dielectrophoretic forces between parallel, open surface, and transparent Indium Tin Oxide (ITO) electrodes for the manipulation and nanoscale trapping of DNA. Unlike other approaches, our method leverage the benefits of nanoscale confinement and nanoscale manufacturing methodologies to provide a new means to confine DNA molecules within a single field of view. Our technology allows trapping, confinement, extension, and optical observation of single molecules within an open surface without requiring mechanical components or a thin (nanoscale) vertical device dimension. In particular, eliminating the requirement of having a thin vertical device dimension obviates the need for direct bonding and the need for applying high pressure to load DNA into highly confined device features. To this end, this technology provides a new method for single-molecule trapping and optical analysis.

## Methods

**Figure 11:**
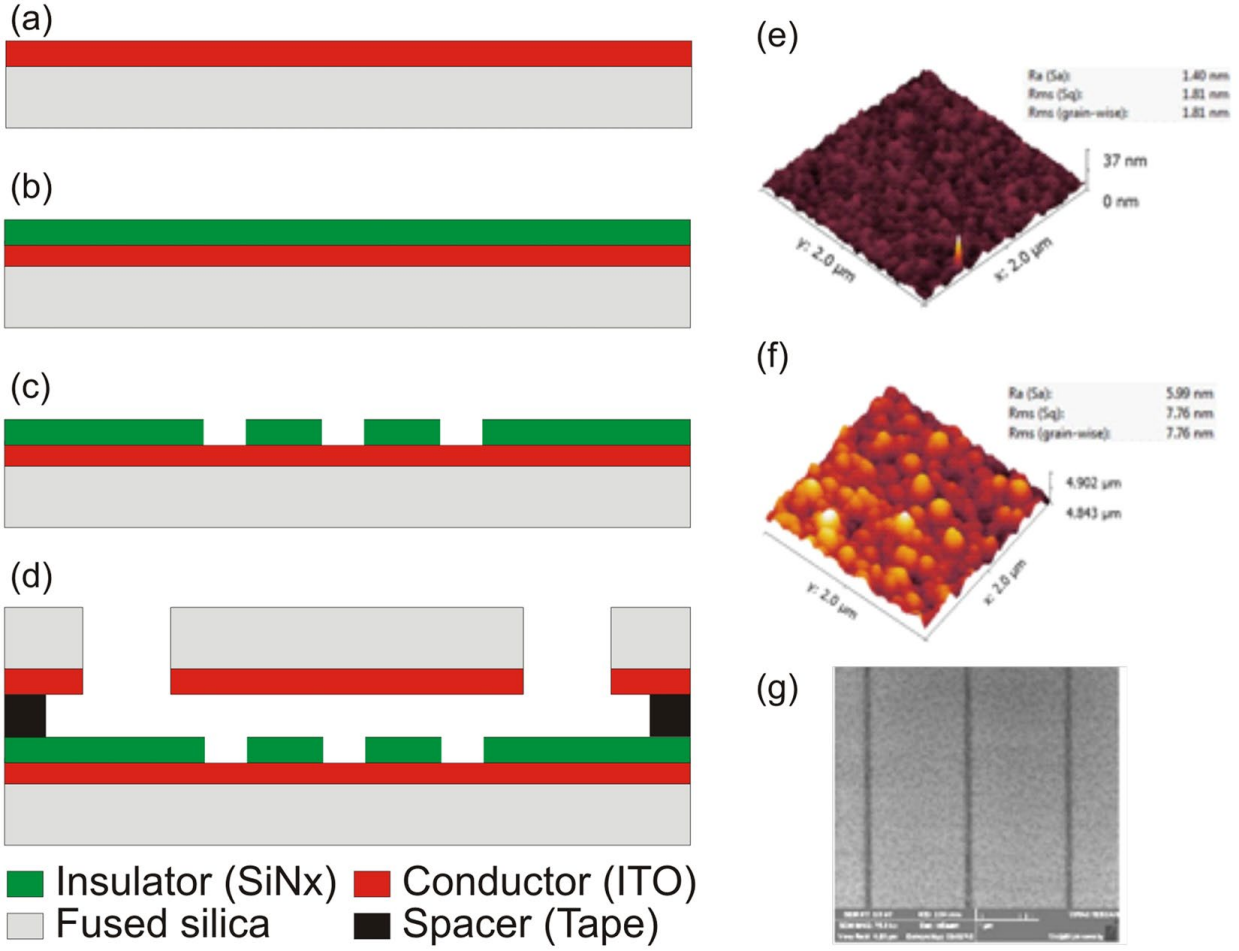
Fabrication flow (**a**) transparent conductive layer (Indium Tin Oxide) coating on fused silica substrate, (**b**) deposition of insulator layer, (**c**) reactive ion etching of insulator layer using E-beam lithography mask, (**d)** formation of flow chamber by joining top and bottom layer with double-sided tape, (**e**,**f**) AFM surface morphologies of electrode surface and insulator (SiNx) surface showing smooth ITO surface with roughness RMS of 1.81 nm, and (**g**) SEM image of fabricated nanochannel on the insulator layer.

In our method, the device contains a top cover slip coated with transparent conductive material (Indium Tin Oxide, ITO) and a bottom surface consisting of patterned nano topographies on a SiNx (Silicon Nitride) substrate with blanket-coated transparent conductive material. The top and bottom surfaces are coupled with a micro-scale double-sided laser-cut tape that defines the flow cell. With application of an alternating (AC) electric field (e.g., 2 V_p-p_) between the top and bottom electrodes, a DEP field gradient is established. The micro and nanostructures are patterned on an insulator layer (SiNx), which is coated on a transparent Indium Tin Oxide- (ITO) coated glass. A 30-μm double-sided tape separates the bottom and top layers. The fabrication process steps (Figure 11) and fabrication methodology are described below.

### Fabrication

Fabrication begins by RCA cleaning of a fused silica substrate wafer. The substrate layer is then coated with a conductive layer (Indium Tin Oxide, ITO). A thin layer of ITO (100 nm) is sputtered on the fused silica substrate from the InSn target using an RF sputter tool (Figure 11a). The silicon nitride (SiN_x_) insulator layer is then deposited using a chemical vapour deposition method with SiH_4_-, N_2_-, and NH_3_-based chemistry using a PECVD (P5000) system (Figure 11b). The microscale electrodes are defined via UV lithography and dry etched into a silicon nitride (SiNx) layer via CF_4_CHF_3_-reactive ion etching (RIE P5000). The electrodes are 10 to 50 μm wide. The etching duration is determined in a way that ensures the patterned SiNx layer is thoroughly etched and the ITO electrode underneath is exposed. The features below 1 μm, including nanochannels and nanopits (50 nm to 1 μm), were patterned on the insulator layer via Electron Beam Lithography (EBL) followed by dry RIE etching (Figure 11c). The top layer is fabricated by blanket coating of ITO on a fused silica cover slip (Figure 11a). Finally, the top and bottom layers were attached with patterned tape to define the space between the top and bottom layers. A 30-μm double-sided tape separates the bottom electrode, with its patterned ITO-SiNx features, from the top ITO-coated coverslip (Figure 11d). The tape is laser-cut to create channels to allow liquid to flow into a central microfluidic chamber containing the patterned ITO-SiNx features. Small holes are sand-blasted into the corners of the device for fluid injection and buffer exchange. The fluid containing the DNA sample is then pipetted into the chip via sand-blasted holes on the top cover slip.

Fabrication accuracy and integrity were investigated using SEM and AFM imaging and surface morphologies. The surface roughness of the etched bottom surface is important. A rough surface may result in DNA molecules sticking to the channel. Therefore, roughness of the bottom channel surface was measured using Atomic Force Microscopy (AFM) scans on the surfaces. According to the AFM results, the ITO electrode possesses a roughness RMS of 1.8 nm (Figure 11e). A rough insulator surface may also make DNA stick onto the surface so we also investigated the top surface using AFM surface scans. The AFM results showed a smooth insulator surface with an RMS of 7.76 nm (Figure 11f). A SEM image of the nanochannel on the insulator layer is shown in Figure 11g.

### Microscopy, chemicals and setup

The imaging of fluorescent DNA molecules was performed on a Nikon Ti-E inverted microscope equipped with a Nikon 100x oil-immersion objective (Nikon CFI Apo 100XW NIR) and an Andor iXon Ultra EMCCD camera. The conductive (ITO) electrodes were connected to an AC power supply through a conductive path made of thin copper tape. After mounting the device on the microscope stage, the DNA solution containing YOYO1 stained Lambda-Phage DNA is inserted into the flow cell. The DNAs used in this experiment were λ-Phage DNA (48.5 kbp), which was stained for visualization with YOYO-1, at a 10:1 intercalation ratio. YOYO-1 is known to increase the full contour length of DNA; for example, at this staining ratio from 16.5 μm to 19.07 μm for λ-phage DNA. The buffer used in this experiment was 1x TBE, which is a solution of Tris-base, boric acid and EDTA (ethylenediaminetetraacetic acid). DNA was used at a concentration of 50 mg/mL. Occasionally, 3% (vol/vol) Beta-mercaptoethanol BME) was added as an anti-photobleaching agent.

In the DEP experiment, frequency and voltage are adjusted with a power supply and frequency generator. The input signal was continuously recorded using an oscilloscope. Before applying voltage, the YOYO1-stained DNA molecules were observed under a fluorescent microscope. After applying voltage at small frequencies (>1 KHz), DNA molecules start moving inside the gap between the top and the bottom electrodes.

## Electronic supplementary material

DNA linearization
